# Pathological Anatomy of the Brain, Spinal Cord, and Their Membranes, with 13 Coloured Plates

**Published:** 1831

**Authors:** 


### LXIV.

### PATHOLOGICAL ANATOMY OF THE BRAIN, SPINAL CORD, AND THEIR MEMBRANES, with 13 coloured plates. By W. P. COCKS. Octodecimo.—Highley, 1831.

THIS is really a very meritorious little pocket-companion, condensing in a very small space, the essence of our knowledge, up to the present time, respecting the morbid anatomy of the parts above-mentioned. "The motive which led to this undertaking, was the great expense of coloured drawings on subjects essentially necessary for the student, and it is hoped that these plates, coloured after Nature, may be refreshing to the memory even of the more advanced inquirer. It frequently happens that the best written descriptions fall short of that power to identify which the pencil conveys, and with this feeling, the author, in the prosecution of his pathological researches, has made it his practice, for years past, not only to take written notes of all his post-mortem examinations, but sketches also of every change in structure that has appeared at all remarkable or unusual."

We have not seen any little vade mecum of this kind so well calculated to fulfil the design of the author as this small volume of Mr. Cocks.

